# RNAi Pathway Genes Are Resistant to Small RNA Mediated Gene Silencing in the Protozoan Parasite *Entamoeba histolytica*

**DOI:** 10.1371/journal.pone.0106477

**Published:** 2014-09-08

**Authors:** Justine M. Pompey, Laura Morf, Upinder Singh

**Affiliations:** 1 Department of Microbiology and Immunology, Stanford University School of Medicine, Stanford, California, United States of America; 2 Division of Infectious Diseases, Department of Internal Medicine, Stanford University School of Medicine, Stanford, California, United States of America; Wuhan University, China

## Abstract

The RNA interference pathway in the protist *Entamoeba histolytica* plays important roles in permanent gene silencing as well as in the regulation of virulence determinants. Recently, a novel RNA interference (RNAi)-based silencing technique was developed in this parasite that uses a gene endogenously silenced by small RNAs as a “trigger” to induce silencing of other genes that are fused to it. Fusion to a trigger gene induces the production of gene-specific antisense small RNAs, resulting in robust and permanent silencing of the cognate gene. This approach has silenced multiple genes including those involved in virulence and transcriptional regulation. We now demonstrate that all tested genes of the amebic RNAi pathway are unable to be silenced using the trigger approach, including Argonaute genes (Ago2-1, Ago2-2, and Ago2-3), RNaseIII, and RNA-dependent RNA polymerase (RdRP). In all situations (except for RdRP), fusion to a trigger successfully induces production of gene-specific antisense small RNAs to the cognate gene. These small RNAs are capable of silencing a target gene in trans, indicating that they are functional; despite this, however, they cannot silence the RNAi pathway genes. Interestingly, when a trigger is fused to RdRP, small RNA induction to RdRP does not occur, a unique phenotype hinting that either RdRP is highly resistant to being a target of small RNAs or that small RNA generation may be controlled by RdRP. The inability of the small RNA pathway to silence RNAi genes in *E. histolytica*, despite the generation of functional small RNAs to these loci suggest that epigenetic factors may protect certain genomic loci and thus determine susceptibility to small RNA mediated silencing.

## Introduction

RNA interference (RNAi) is the regulation of gene expression through small (∼20–30 nucleotide), noncoding RNAs that target transcripts for silencing in a sequence-specific manner [1]. RNAi mechanisms have been identified in a wide array of organisms from protozoans to humans and small RNAs (sRNAs) function in diverse biological processes from development to antiviral defense [2]–[6]. In the classical RNAi pathway, double-stranded RNA (dsRNA) is recognized and cleaved by Dicer, an RNaseIII enzyme, into 20-30nt duplexes that get loaded into an RNA-Induced Silencing Complex (RISC). One strand is preferentially degraded, activating RISC to use the other strand to target transcripts with which it has perfect or near perfect complementarity for transcriptional or post-transcriptional silencing [7]–[9]. Argonaute (AGO) is a key component of RISC and is responsible for mediating silencing using the target sRNA [9]. RNAi-mediated silencing can take many forms including transcript cleavage, transcriptional repression, and DNA and histone modifications [9]. In some organisms, such as *Schizosaccharomyces pombe*, RNAi is required for heterochromatin formation [10].

In nematodes a secondary RNAi pathway functions, in which RNA-dependent RNA Polymerase (RdRP) is recruited to sites of primary RNAi and synthesizes secondary sRNAs resulting in amplified silencing [7], [11], [12]. Many classes of sRNAs including small interfering RNAs (siRNAs) and microRNAs are processed through an RNaseIII-dependent mechanism, which generates sRNAs with 5′-monophosphate and 3′-hydroxyl termini [13]. In contrast, RdRP-generated secondary sRNAs in nematodes contain 5′-triphosphate termini [11], [12], [14]. Excluding nematodes, sRNAs with 5′-polyphosphates (5′-polyP) have only been described in one other system, the protozoan parasite *Entamoeba histolytica* (*E. histolytica*) [15]–[17].

*E. histolytica* is an important human intestinal parasite that results in 100,000 deaths annually and is thus a leading parasitic cause of death. Invasive disease is characterized by dysentery, colitis, and abscesses in the liver [18], [19]. Gene expression regulation modulates many aspects of parasite pathogenesis including tissue invasion and response to oxidative stress [20], [21]. A robust endogenous small RNA pathway is present in *E. histolytica* [22]. Core elements of the RNAi machinery are encoded within the *E. histolytica* genome including three Argonaute genes (EhAgo2-1, EhAgo2-2, and EhAgo2-3) (EHI_186850, EHI_125650, EHI_177170) and two RdRP genes (EhRdRP1 and EhRdRP2) (EHI_139420 and EHI_179800) [23]. To date, no canonical Dicer enzyme in *E. histolytica* has been identified, however there is a single gene with an RNaseIII domain (EhRNaseIII) (EHI_068740) annotated in the genome [23]. Of the three Argonaute genes, EhAgo2-2 is the most highly expressed [23], [24]. Although there are two RdRP genes in *E. histolytica*, only EhRdRP1 contains a full-length RdRP domain [23].

*E. histolytica* has a complex repertoire of endogenous sRNAs including a highly abundant 27nt population, which associates with EhAGO2-2 and has 5′-polyP termini indicating that they are not Dicer products but instead are reminiscent of secondary sRNAs in nematodes [11], [12],[14],[15],[17]. Among the EhAGO2-2 associated sRNAs, the majority map antisense (AS) to predicted protein coding regions, are biased towards the 5′ end of the gene, and have an inverse correlation between sRNA abundance and gene expression indicating that they regulate gene expression in *E. histolytica* [15], [17]. Comparisons between *E. histolytica* strains suggest that sRNAs regulate virulence factors that contribute to strain-specific differences [17]. Furthermore, sRNAs help mediate transcriptional gene silencing in *E. histolytica* [16].

The polyploid genome and lack of homologous recombination make traditional gene knockout strategies unfeasible in *E. histolytica* and have led to alternative approaches to down regulating gene expression [25], [26]. Most of these methods are based on the RNAi pathway and include expression of AS transcripts, dsRNA hairpins, short RNA hairpins, soaking trophozoites in siRNAs, and feeding dsRNA-expressing bacteria to trophozoites [reviewed in [27]]. These techniques exhibit varying degrees of success and further investigations into the underlying silencing mechanism have been largely lacking [27]. Moreover, there is evidence that dsRNA-based silencing can be lost over time [28]. A method of transcriptional gene silencing (termed G3) in which chromatin modifications at the genomic locus lead to permanent silencing was recently linked to the RNAi pathway through the involvement of EhAGO2-2 and sRNAs to silenced loci [16].

Recently a technique was developed that takes advantage of the endogenous RNAi pathway to silence genes in *E. histolytica* using a mechanistic approach [29]. This method uses a “trigger” gene (which has a dense population of endogenous AS sRNAs) that when fused to a second gene silences the fused gene [29]. Silencing occurs via generation of AS sRNAs to the fused gene and targeting of the chromosomal gene resulting in silencing. Importantly, even after subsequent removal of the trigger plasmid, there is continued amplification of the AS sRNAs from the chromosomal locus enabling permanent silencing [29]. This technique has successfully silenced multiple *E. histolytica* genes [29], [30].

Given the robust down regulation of *E. histolytica* transcripts using the novel trigger-based approach, we wanted to ascertain whether this technique could silence RNAi genes in *E. histolytica*. Targeting RNAi pathway genes for silencing using an RNAi-based method has been successful in other systems [31]–[33]. Using the trigger-based approach we attempted to silence three Argonaute genes (EhAgo2-1, EhAgo2-2, EhAgo2-3), EhRNaseIII, and EhRdRP1. For the Ago and RNaseIII genes, the trigger method generated gene-specific AS sRNAs, but despite this, all genes were refractory to silencing. For EhRdRP1 no AS sRNAs could be detected in parasites with the trigger-based approach. Given that none of the amebic RNAi genes could be downregulated using the trigger method, these data raise the possibility that the genomic loci of the putative RNAi genes are protected from RNAi-mediated silencing.

## Results

### Trigger mediated generation of small RNAs to EhAgo2-2

**Figure 1:**
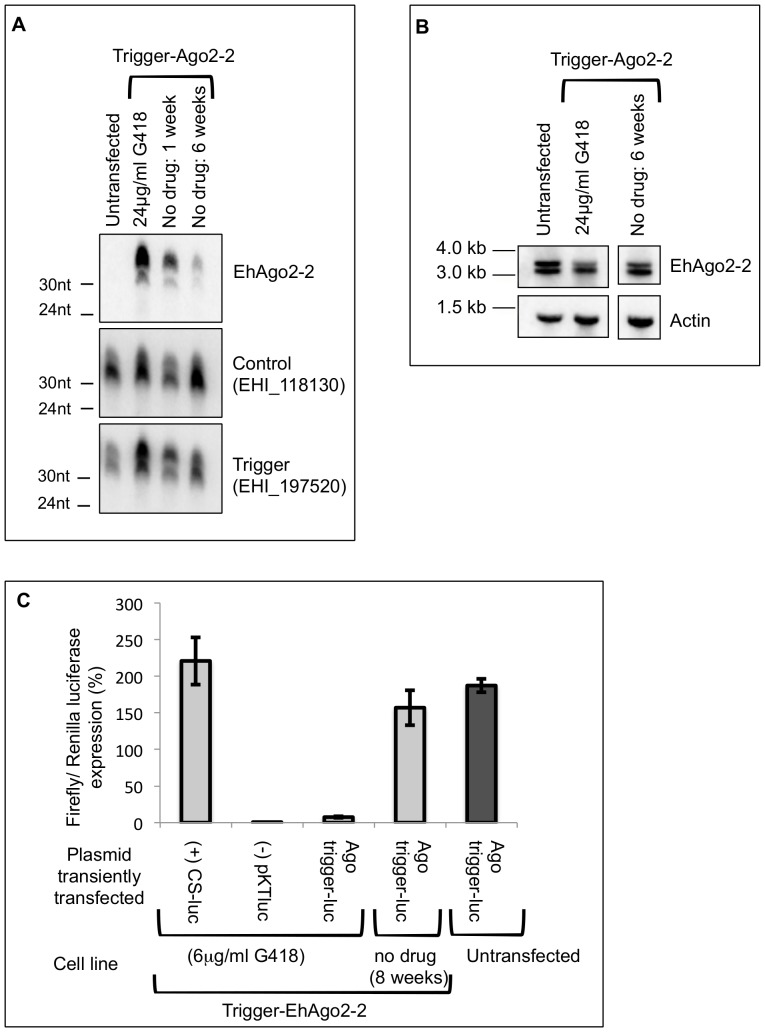
Antisense sRNAs to EhAgo2-2 are functional but do not silence gene. (**A**) High resolution Northern blot of the trigger-EhAgo2-2 cell line probed for AS sRNAs to EhAgo2-2, EHI_188130 (loading control), and EHI_197520 (trigger) at 24 µg/ml drug and at 1 and 4 weeks without drug. Abundant AS sRNAs to EhAgo2-2 diminish over time with plasmid removal. (**B**) Northern blot for EhAgo2-2 transcripts in untransfected parasites and in the trigger-EhAgo2-2 cell line at 24 µg/ml drug and at 6 weeks post drug removal. (**C**) Luciferase assays in the trigger-EhAgo2-2 cell line transiently transfected with a luciferase expression plasmid (CS-luc), a promoter-less luciferase plasmid (pKTluc), or with the Ago2-2 trigger-luciferase plasmid. Parasites were maintained at 6 µg/ml drug or were removed from drug for 8 weeks. Luciferase expression was inhibited in the presence of an Ago2-2 trigger and restored when trigger-EhAgo2-2 plasmid was lost. Luciferase values were normalized to the CS-Renilla-luc expression. Experiments were performed in triplicates; average and standard errors are shown.

We have previously tried a variety of methods (including expression of inducible and constitutive AS transcripts and dominant negative strategies) to modulate expression levels of the core RNAi machinery in *E. histolytica* but have been unable to downregulate these genes in *E. histolytica* trophozoites (Pompey and Singh, unpublished data). With the recent development of a novel and robust trigger silencing method, we wanted to determine whether this approach was capable of silencing amebic RNAi genes. We attempted to silence EhAgo2-2 using the full-length coding sequence of EhAgo2-2 cloned downstream of a trigger plasmid. High resolution Northern blot analysis using strand-specific oligonucleotide probes revealed abundant gene-specific AS sRNAs to EhAgo2-2 in trophozoites expressing the trigger-EhAgo2-2 plasmid but not in untransfected parasites (Figure 1A). Similarly, AS sRNAs to the trigger region were enriched in transfected parasites confirming that sRNA amplification was occurring from the trigger plasmid as expected. These results agree with previously published results showing that AS sRNAs can be generated to endogenous *E. histolytica* genes fused to the trigger [29]. To assess impact on EhAgo2-2 transcript levels, we probed for full-length transcript by Northern blot analysis. There was a slight decrease in the abundance of EhAgo2-2 transcripts in parasites transfected with the silencing construct compared to those that were not (Figure 1B) indicating that the Ago2-2 AS sRNAs were not able to efficiently silence the EhAgo2-2 transcripts. These data are in sharp contrast to other *E. histolytica* genes whose transcripts were silenced to below the levels of detection using this trigger method [29]. Similar strategies have been employed in other systems including *Drosophila*, *Caenorhabditis elegans*, and *Trypanosoma brucei* where core RNAi genes could be successfully downregulated using an RNAi-based approach [31]–[33].

### Trigger generated small RNAs are not maintained in the absence of drug selection

Morf et al. [29] demonstrated that AS sRNAs to endogenous genes were maintained even after removal of the silencing plasmid, indicating that the chromosomal locus was serving as a template for the amplification of sRNAs originally induced by the trigger plasmid. To determine whether EhAgo2-2 sRNAs could enter an amplification process based on a chromosomal template, we induced the loss of the trigger plasmid from the transfectants by continuously culturing parasites in absence of drug pressure for six weeks and tested for maintenance of the EhAgo2-2 AS sRNAs by Northern blot analysis. After one week without drug, sRNAs to EhAgo2-2 were still abundant but after drug removal for six weeks, the population of EhAgo2-2 sRNAs was substantially diminished (Figure 1A). This result is in stark contrast to the trigger-mediated silencing of the *E. histolytica* rhomboid protease EhROM1 where EhROM1 sRNAs were abundant even after 18 months of culturing without drug [29]. This indicates that unlike EhROM1, EhAgo2-2 sRNAs are not efficiently maintained by an amplification process generated at the genomic locus. Thus it appears that some loci are amenable to initiating an amplification loop and long-term maintenance of sRNAs, while others are not. Whether there are epigenetic differences between the two loci that could be responsible for the observed differences in sRNA maintenance remain to be determined.

### Trigger generated small RNAs to Ago2-2 are functional

Given that there was no substantial silencing of EhAgo2-2 despite the presence of AS sRNAs to this gene, we wanted to determine whether the Ago2-2 sRNAs were functional. We have previously observed that in cell lines with sRNAs induced by the trigger method, the sRNAs function in trans and can silence a new target [29]. Therefore, to probe the ability of EhAgo2-2 sRNAs to silence a gene, we used the first 132bp of EhAgo2-2 as a “trigger” and fused it to a luciferase reporter generating the Ago trigger-luc construct. We then transiently transfected this plasmid into the trigger-EhAgo2-2 cell line, which contained abundant EhAgo2-2 AS sRNAs, and assessed for luciferase signal. As a positive control, the trigger-EhAgo2-2 parasites were transiently transfected with a construct (CS-luc) containing luciferase driven by the strong cysteine synthase promoter. Trophozoites transfected with a promoter-less construct (pKTluc) served as negative controls. Both the positive and negative control functioned as expected and high luciferase expression was observed with the CS-luc construct but no luciferase signal was detected with the construct lacking the promoter (Figure 1C). However, luciferase signal was not detected in parasites transfected with the Ago trigger-luc construct indicating that the original trigger-generated EhAgo2-2 AS sRNAs were functional in trans and capable of efficient silencing of the fused luciferase gene (Figure 1C). In contrast, when EhAgo2-2 sRNA levels were low after removal from drug pressure, an exogenously transfected CS-luciferase was expressed at levels similar to wild-type parasites (Figure 1C). The ability of the EhAgo2-2 sRNAs to silence an exogenously introduced plasmid in trans, indicates that these sRNAs are functional and it is unclear why EhAgo2-2 sRNAs are unable to silence the cognate gene. We hypothesize that some inherent feature of EhAgo2-2 genomic locus prohibits silencing.

### Antisense sRNAs are generated to putative amebic RNAi genes but do not silence targets

**Figure 2:**
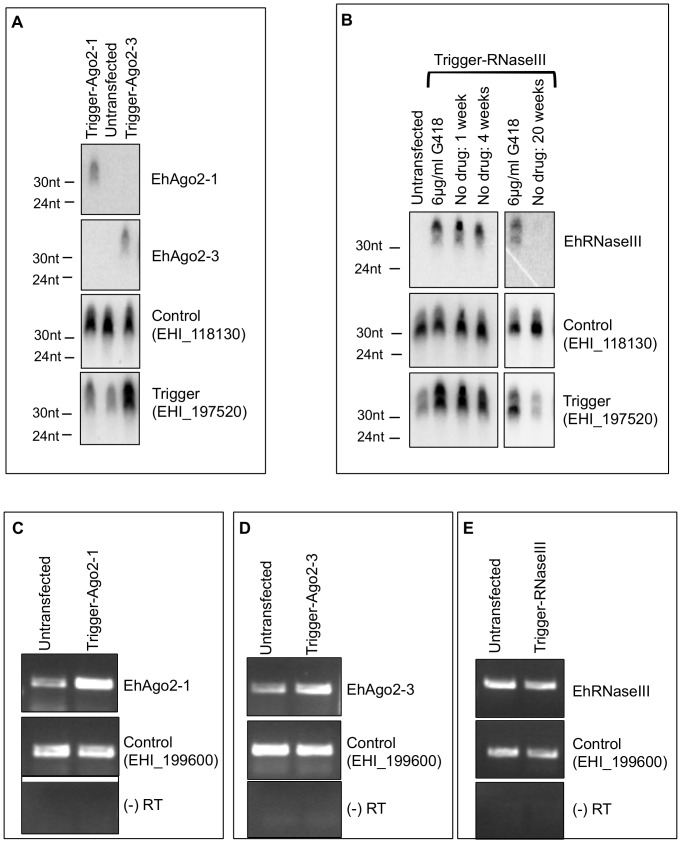
Antisense sRNAs are detected to putative RNAi genes but do not silence the target genes. (**A**) High resolution Northern blot of parasites transfected with the trigger-EhAgo2-1 or the trigger-EhAgo2-3 plasmid. Cell lines maintained at 24 µg/ml G418. Gene-specific AS sRNAs to Ago2-1 or Ago2-3 are detected in the respective cell lines. AS sRNAs to EHI_188130 (loading control) and EHI_197520 (trigger) serve as controls. (**B**) High resolution Northern blot analysis of trigger-EhRNaseIII transfectants maintained in 6 µg/ml G418 or removed from drug selection for 1, 4, or 20 weeks. Abundant EhRNaseIII AS sRNAs are detected at all time points excluding the 20-week removal from drug sample. The 20-week sample shows a very faint population of EhRNaseIII AS sRNAs. Controls are the same as in (A). (**C**) Semi-quantitative RT-PCR of trigger-EhAgo2-1 transfectants at 24 µg/ml G418 shows an increase in EhAgo2-1 transcript abundance compared to untransfected cells. EHI_199600 was used as a loading control and -RT samples as negative controls. (**D**) Semi-quantitative RT-PCR shows an upregulation of EhAgo2-3 transcript in the trigger-EhAgo2-3 cell line at 24 µg/ml G418 compared to untransfected cells. Controls are the same as in (C). (**E**) Semi-quantitative RT-PCR of trigger-EhRNaseIII transfectants at 6 µg/ml G418 shows that the abundance of EhRNaseIII transcript is unaffected by the presence of gene-specific AS sRNAs. Controls are the same as in (C).

In addition to EhAGO2-2, there are several other members of the RNAi pathway encoded within the *E. histolytica* genome including two additional Argonautes, EhAGO2-1 and EhAGO2-3, and an RNaseIII enzyme, EhRNaseIII [23]. There is no clear Dicer enzyme in *E. histolytica*; however, EhRNaseIII is the only RNaseIII domain-containing gene annotated in the genome [23]. To determine whether these genes could be silenced using the trigger approach, we fused each gene to the trigger construct and generated stable transfectants. We assayed for AS sRNA generation to the gene of interest, to a control gene with abundant sRNAs (EHI_188130), and to the trigger region by Northern blot analysis. Antisense sRNAs to EhAgo2-1 and EhAgo2-3 were detected in their respective cell lines, and sRNAs to the trigger region were enriched in both the EhAgo2-1 and EhAgo2-3 cell lines compared to untransfected parasites (Figure 2A).

Similar results were obtained with EhRNaseIII where gene-specific AS sRNAs to EhRNaseIII were detected in transfectants expressing the silencing plasmid (Figure 2B). To ascertain whether EhRNaseIII AS sRNAs could persist after plasmid removal, trophozoites were cultured for 20 weeks in the absence of drug and the presence of AS sRNAs determined by Northern blot analysis. At one and four weeks without drug, the EhRNaseIII sRNA population was readily detectable and the abundance similar to parasites maintained at 6 µg/ml of G418 (Figure 2B). However, after 20 weeks without drug selection, the EhRNaseIII AS sRNA population was nearly undetectable and the trigger sRNAs had returned to wild-type levels (Figure 2B). Collectively, these data indicate that EhRNaseIII AS sRNAs may persist for a short term, but that they do not persist for long periods after plasmid removal. This suggests that the endogenous EhRNaseIII locus is not an efficient template for long-term sRNA maintenance. Similar to EhAgo2-2, these results are in sharp contrast to other amebic genes tested in this system, where sRNAs to targeted genes are maintained long-term even after the trigger plasmid is lost [29].

To determine whether generation of AS sRNAs affected transcript levels for EhAgo2-1, EhAgo2-3 and EhRNaseIII, we performed reverse transcriptase polymerase chain reaction (RT-PCR) for each gene. In the trigger-EhAgo2-1 cell line the abundance of the EhAgo2-1 transcript was increased compared to untransfected parasites (Figure 2C); similar results were observed with EhAgo2-3 in the trigger-EhAgo2-3 transfectants (Figure 2D). Thus, it appears that the Ago transcripts are not silenced by the trigger approach as has been seen previously with EhROM1, EhMyb, and EhHRM-BP [29], [30]. When we compared EhRNaseIII transcript in trigger-EhRNaseIII parasites to untransfected cells, there was no substantial decrease in transcript abundance (Figure 2E). Thus, the RNAi genes – EhAgo2-1, EhAgo2-2, EhAgo2-3, and EhRNaseIII - are all refractory to silencing utilizing this system despite the generation of gene-specific AS sRNAs. The underlying basis for this phenomenon remains to be elucidated.

### Trigger silencing approach unable to generate antisense sRNAs to EhRdRP1

**Figure 3:**
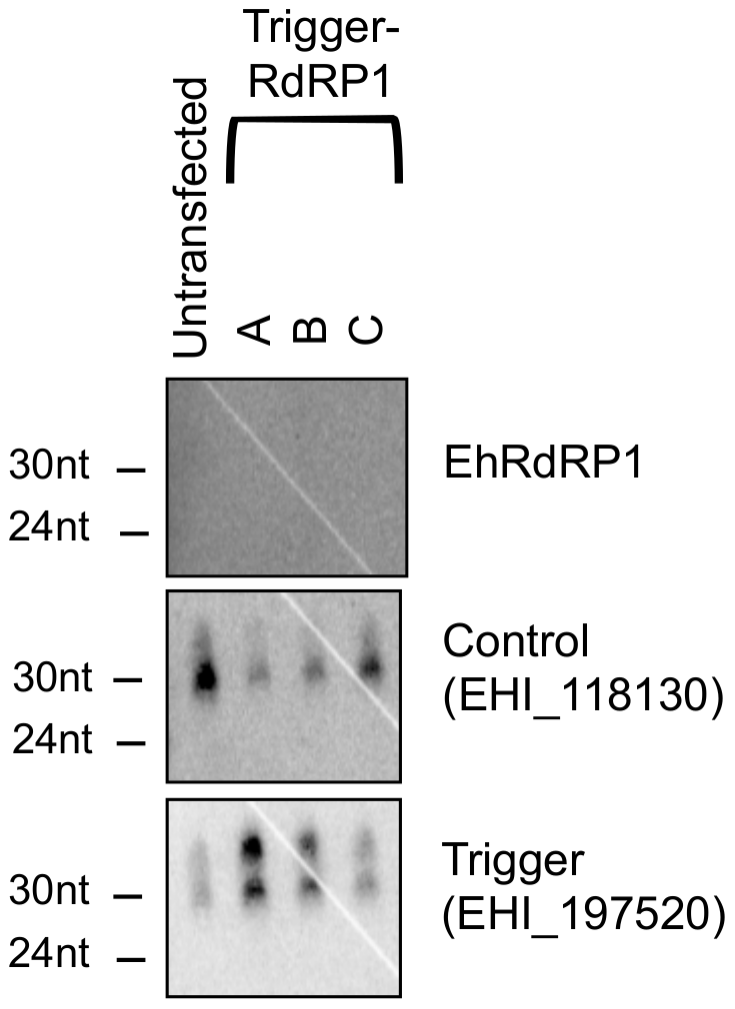
The fusion of the trigger to EhRdRP1 does not result in generation of AS sRNAs to EhRdRP1. High resolution Northern blot of trophozoites transfected with the trigger-EhRdRP1 plasmid. The lines A, B, and C, are three independently transfected cell lines maintained at 24 µg/ml G418. No RdRP1 AS sRNAs were detected in 75 µg of small-RNA enriched material from any of the three transfectant cell lines. EHI_118130 and EHI_197520 serve as controls.

The trigger sRNAs contain 5′-polyP termini indicating that they are generated in a Dicer-independent manner [29], [34]. Small RNAs with similar 5′ structures are found in other systems, where they are generated by RdRP [11], [12], [14]. To determine whether EhRdRP1, as a putative member of the amebic RNAi pathway, was amenable to silencing using the trigger method, we generated transfectants expressing the trigger-EhRdRP1 plasmid and probed for generation of EhRdRP1-specific AS sRNAs by Northern blot analysis. We could not detect sRNAs to EhRdRP1 in three independently generated transfectant lines (Figure 3). To address the possibility that sRNAs to EhRdRP1 could be less abundant, we did Northern blot analysis with 100 µg of sRNA enriched material and tried multiple probes to EhRdRP1. However, these conditions failed to reveal EhRdRP1-specific AS sRNAs in the transfected cell lines (data not shown). Although sRNAs to EhRdRP1 were not detected in the trigger-EhRdRP1 transfectants, the initial trigger AS sRNAs were enriched (Figure 3) indicating that trigger sRNAs were being amplified from the plasmid. This outcome is unique among the putative RNAi genes tested here as well as among the other amebic genes tested previously [29], [30]. These data suggest that there is something specific about EhRdRP1 that is prohibiting the generation of specific sRNAs to this gene and may have implications for the biogenesis of sRNAs in *E. histolytica*.

## Discussion

The recent development of a trigger-based sRNA silencing method demonstrates how the endogenous RNAi pathway can be used as a tool to regulate the expression of specific genes in *E. histolytica* [29], [30]. A trigger sequence, to which abundant endogenous AS sRNAs map, can silence genes fused to it through the induction of gene-specific AS sRNAs. In this approach, both the episomal and chromosomal gene copies are silenced and silencing is maintained despite loss of the trigger plasmid [29]. In this study, we applied the trigger silencing approach to modulate expression of RNAi machinery genes. Our data demonstrate that for the majority of RNAi genes the trigger approach successfully generates functional AS sRNAs to the gene of interest, yet gene expression is not downregulated. Additionally, there is limited sustained amplification from the chromosomal locus, which is in contrast to previous results. These data raise interesting questions about the nature of the genomic loci of core RNAi machinery in *E. histolytica* and whether epigenetic modifications could play a role in susceptibility or resistance to RNAi-based silencing.

Despite not silencing the EhAgo2-2 locus, the trigger-sRNA method was able to generate AS sRNAs to EhAgo2-2, which were functional in trans. It is unclear why the Argonaute and RNaseIII genes are refractory to silencing with this method, however one possibility is that these genes are essential for parasite viability and thus the parasite circumvents silencing to ensure their continued expression. That EhAgo2-1 and EhAgo2-3 transcripts were upregulated in parasites containing the silencing construct compared to untransfected parasites may support this hypothesis. It is possible that the genes that fuel the RNAi pathway in *E. histolytica* are unable to be downregulated using an RNAi-based approach because they are necessary to produce the silencing phenomenon, although targeting RNAi genes using an RNAi-based method has proven successful in other systems [31]–[33]. A third explanation is that epigenetic determinants or chromatin modifications may render the genomic loci of RNAi genes inaccessible to sRNAs and/or the proteins needed for subsequent processing. Additional support for this hypothesis is the inability of the amplification cascade, which maintains sRNAs despite loss of the trigger plasmid, to successfully retain sRNAs to EhAgo2-2 and EhRNaseIII. Thus, modifications of the genomic loci may exist which make it inaccessible for RNAi-mediated silencing. Alternatively, perhaps *E. histolytica* encodes RNA silencing suppressors that uniquely target the RNAi machinery. Many viruses employ similar strategies as an antiviral defense mechanism within their hosts [35].

Another interesting aspect of our data is that sRNAs generated to RNAi genes are not efficiently maintained upon plasmid removal. In contrast, when the trigger method targets other genes, sRNAs persist and are detectable for more than a year after removal of the trigger silencing plasmid [29]. In the *E. histolytica* G3 silencing system, which mediates transcriptional gene silencing through AS sRNAs and histone modifications, similar long-term maintenance of sRNAs is noted [16]. This suggests that the chromosomal locus of the targeted gene serves as a template for continued sRNA generation [29]. The lack of long-term sRNA maintenance for RNAi genes could point to differences between the chromosomal loci of the RNAi genes and other endogenous genes for which permanent silencing is possible. In many systems, noncoding RNAs and the RNAi pathway are involved in epigenetic modifications such as inducing *de novo* methylation in plants and formation of heterochromatin in *S. pombe* and *Tetrahymena thermophila* [10], [36]–[40]. In *E. histolytica*, a variety of chromatin modifying enzymes have been described including histone acetyltransferases, a histone deacetylase, and a DNA methyltransferase [41], [42], and enrichment in histone H3 and a decrease in methylation of H3-lysine 4 have been associated with silencing in G3 trophozoites [16], [43]. Therefore, investigating the connection between noncoding RNAs and DNA or chromatin modifications and the potential role chromatin-modifying proteins may play in sRNA mediated silencing in *E. histolytica* is of great interest. It is also likely that as the trigger approach becomes more widely utilized, additional resistant loci will be identified and future studies will need to focus on identifying the epigenetic characteristics of resistant loci.

The outcome with targeting the EhRdRP1 gene using the trigger approach is unique since sRNAs to EhRdRP1 were not detected using the trigger approach. A simple explanation is that sRNAs are generated but are of low abundance so as not detectable by Northern blot methods. Antisense sRNAs produced by the trigger system contain 5′-polyP termini reminiscent of secondary sRNAs in *C. elegans* [11], [29] suggesting that these AS sRNAs may be generated by an RdRP enzyme. Thus, given the role of nematode RdRPs in generating secondary sRNAs with 5′-triphosphosphate termini [11], [12], [14], a more intriguing possibility is that the inability of the trigger to generate AS sRNAs to RdRP is due to the absolute requirement of RdRP for this process. However, further work is needed to verify this hypothesis.

**Figure 4:**
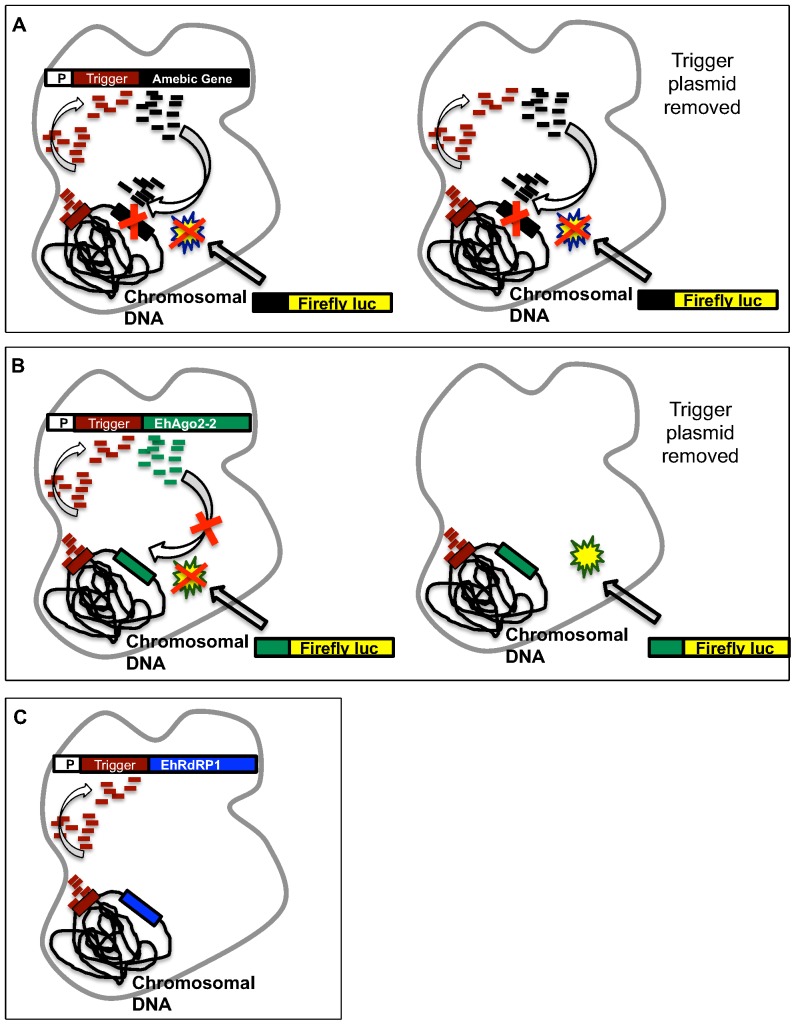
Schematic of model. (**A**) Functional AS sRNAs are generated to some amebic genes when fused to the trigger and the episomal and endogenous genes are silenced. sRNAs and silencing persist after plasmid removal and silencing can be extended to a fusion construct containing a portion of the endogenous gene. (Modified from [29]). (**B**) Functional AS sRNAs to some amebic RNAi genes are induced when fused to the trigger but the endogenous gene is not silenced. Amebic RNAi sRNAs are not maintained after plasmid removal. (**C**) No AS sRNAs are made to EhRdRP1, and therefore EhRdRP1 is not silenced.

We have shown that the genes that fuel the RNAi pathway in *E. histolytica* are unable to be downregulated using the RNAi-based trigger approach. We hypothesize that the point at which the trigger approach “stalls” is related to the putative role of each gene in the RNAi pathway. Thus in our working model, AS sRNAs are produced to amebic Argonaute genes because they function downstream of 5′-polyP sRNA generation (Figure 4). The Argonaute targeted sRNAs are functional as they can serve as triggers to induce silencing of an exogenous gene; however, these sRNAs are incapable of silencing their chromosomally encoded cognate gene as the genomic locus appears inaccessible to sRNA-mediated effects, perhaps due to its chromatin structure. In contrast, because RdRP may be responsible for generating AS sRNAs, it is recognized as essential by the parasite and is absolutely protected from AS sRNA generation.

This work demonstrates that although the RNAi pathway is a useful tool for genetic manipulation, the genes that fuel this pathway in *E. histolytica* cannot be silenced using this method. Interesting questions have been raised about possible epigenetic differences between susceptible and resistant loci for targeting by RNAi in *E. histolytica*. Some questions of interest are: Are there patterns in epigenetic or chromatin modifications among loci that can be silenced using the trigger method? Do those trends or modifications occur at RNAi gene loci? Similarly, are there epigenetic trends that are unique to the loci of RNAi genes? And what is the epigenetic or chromatin status of RNAi loci of untransfected parasites compared to those expressing abundant AS sRNAs to RNAi genes? Further work is needed to determine if epigenetic differences among susceptible and resistant loci do exist and the nature of those differences.

## Materials and Methods

### Parasite culture, generation of transgenic parasite strains, and RNA extraction

*Entamoeba histolytica* trophozoites (HM-1: IMSS) were grown axenically under standard conditions [24], [44]. Mid-log phase trophozoites were transfected with 20 µg of purified plasmid using 30 µl of Superfect Transfection Reagent (Qiagen) as described in [45]. Stable transfectants were maintained at either 6 µg/ml or 24 µg/ml G418. To induce plasmid loss, transfectants were removed from drug selection and cultured continuously in the absence of G418. RNA was extracted from log phase trophozoites using the small-RNA enriched protocol of the miRVANA kit (Ambion) according to manufacturer's instructions with two modifications: (1) the spin columns were centrifuged at 3,400×g for 20–25 seconds, and (2) RNA was eluted in 600 µl of distilled RNase-free water in three steps. Both the small RNA-enriched and the small RNA-depleted fractions were kept.

### Plasmid construction

All cloning primers used in this study are listed in Table S1 in File S1. Full-length EhAgo2-1 (EHI_186850), EhAgo2-2 (EHI_125650), EhAgo2-3 (EHI_177170), EhRNaseIII (EHI_068740), and EhRdRP1 (EHI_139420) were amplified from *E. histolytica* HM-1: IMSS genomic DNA using forward and reverse primers containing SmaI and XhoI sites, respectively, and cloned into the trigger plasmid described in [29]. This plasmid, based on pKT-3M [46], contains the first 132 bp of EHI_197520 cloned in-frame and immediately downstream of the CS promoter in lieu of the 3xMyc sequence. To generate the Ago-trigger-luc plasmid, the first 132 bp of EhAgo2-2 were amplified and cloned into the AvrII site of the CS-luc plasmid [29] immediately upstream of the firefly luciferase gene.

### RNA isolation, Northern blot analysis, and reverse transcriptase PCR

High resolution Northern blot analysis was performed as published in [15] using 75 µg of small RNA-enriched material. Small RNA samples were resolved on 15% polyacrylamide gels and blots probed using oligonucleotide probes listed in Table S2 in File S1. Northern blots detecting mRNA were performed according to standard protocols [47] using 2 µg of total RNA depleted of small RNAs and probed using PCR probes (PCR primers listed in Table S1 in File S1). Blots were stripped with 1% SDS at 80°C for 20 minutes and re-probed. All primers used for RT-PCR in this study are listed in Table S3 in File S1. RT-PCR was conducted according to standard protocols [47] using 2 µg of RNA of the small RNA-depleted fractions.

### Luciferase assays

Luciferase assays were performed in triplicate as described in [29]. Briefly, parasites were harvested 20–22 hours post-transfection and resuspended in lysis buffer supplemented with protease inhibitors (luciferase assay system, Promega E1500, 1× N-acetyl-L-leucyl-L-leucyl-L-argininal, 1× HALT protein inhibitor cocktail, 1× E-64). Protein concentration was determined by Bradford assay and luciferase activity was measured by a luminometer (Monolight 2010). 30 µg of total protein was added to luciferase reagent (Promega) and relative light units were recorded. For Renilla luciferase activity, the above protocol and the Dual-Luciferase® Reporter Assay System (Promega) protocol were used according to manufacturer's instructions to measure the *Renilla* luciferase after the Firefly luciferase.

## Supporting Information

File S1 Contains the following files: **Table S1. Primers used in plasmid construction in this study.** A list of all primers used in cloning and PCR probe generation are listed. **Table S2. Oligonucleotide probes used in this study.** Northern blot oligonucleotide probes are listed. **Table S3. RT-PCR primers used in this study.** A list of all primers used for RT-PCR.
(DOCX)

## References

[1] Zamore PD, Haley B (2005) Ribo-gnome: The big world of small RNAs. Science 309: 1519–1524. 16141061 10.1126/science.1111444

[2] Carrington JC, Ambros V (2003) Role of microRNAs in plant and animal development. Science 301: 336–338. 12869753 10.1126/science.1085242

[3] Kloc A, Martienssen R (2008) RNAi, heterochromatin and the cell cycle. Trends in Genetics 24: 511–517. 18778867 10.1016/j.tig.2008.08.002

[4] Qu F, Morris TJ (2005) Suppressors of RNA silencing encoded by plant viruses and their role in viral infections. Febs Letters 579: 5958–5964. 16162340 10.1016/j.febslet.2005.08.041

[5] Batista TM, Marques JT (2011) RNAi pathways in parasitic protists and worms. J Proteomics 74: 1504–1514. 21385631 10.1016/j.jprot.2011.02.032

[6] Vance V, Vaucheret H (2001) RNA silencing in plants–defense and counterdefense. Science 292: 2277–2280. 11423650 10.1126/science.1061334

[7] Ghildiyal M, Zamore PD (2009) Small silencing RNAs: an expanding universe. Nat Rev Genet 10: 94–108. 19148191 10.1038/nrg2504 PMC2724769

[8] Sijen T, Vijn I, Rebocho A, van Blokland R, Roelofs D, et al (2001) Transcriptional and posttranscriptional gene silencing are mechanistically related. Current Biology 11: 436–440. 11301254 10.1016/s0960-9822(01)00116-6

[9] Pratt A, Macrae I (2009) The RNA induced silencing complex: A versatile gene-silencing machine. Journal of Biological Chemistry: 1–13. 10.1074/jbc.R900012200 PMC2709356 19342379

[10] Volpe TA, Kidner C, Hall IM, Teng G, Grewal SI, et al (2002) Regulation of heterochromatic silencing and histone H3 lysine-9 methylation by RNAi. Science 297: 1833–1837. 12193640 10.1126/science.1074973

[11] Pak J, Fire A (2007) Distinct populations of primary and secondary effectors during RNAi in C. elegans. Science 315: 241–244. 17124291 10.1126/science.1132839

[12] Sijen T, Steiner FA, Thijssen KL, Plasterk RHA (2007) Secondary siRNAs result from unprimed RNA synthesis and form a distinct class. Science 315: 244–247. 17158288 10.1126/science.1136699

[13] Ji X (2008) The mechanism of RNase III action: how dicer dices. Curr Top Microbiol Immunol 320: 99–116. 18268841 10.1007/978-3-540-75157-1_5

[14] Wang JB, Czech B, Crunk A, Wallace A, Mitreva M, et al (2011) Deep small RNA sequencing from the nematode Ascaris reveals conservation, functional diversification, and novel developmental profiles. Genome Research 21: 1462–1477. 21685128 10.1101/gr.121426.111 PMC3166831

[15] Zhang H, Ehrenkaufer GM, Pompey JM, Hackney JA, Singh U (2008) Small RNAs with 5′-Polyphosphate Termini Associate with a Piwi-Related Protein and Regulate Gene Expression in the Single-Celled Eukaryote *Entamoeba histolytica* . PLoS Pathog 4: e1000219. 19043551 10.1371/journal.ppat.1000219 PMC2582682

[16] Zhang H, Alramini H, Vy T, Singh U (2011) Nucleus-localized Antisense Small RNAs with 5′-Polyphosphate Termini Regulate Long Term Transcriptional Gene Silencing in Entamoeba histolytica G3 Strain. Journal of Biological Chemistry 286: 44467–44479. 22049083 10.1074/jbc.M111.278184 PMC3247957

[17] Zhang H, Ehrenkaufer GM, Hall N, Singh U (2013) Small RNA pyrosequencing in the protozoan parasite Entamoeba histolytica reveals strain-specific small RNAs that target virulence genes. BMC Genomics 14: 53. 23347563 10.1186/1471-2164-14-53 PMC3610107

[18] WHO (1997) A consultation with experts on amoebiasis. Mexico City, Mexico 28029 March 1997. Epidemiological Bulletin. 13–14 p. 9197085

[19] Pritt BS, Clark CG (2008) Amebiasis. Mayo Clinic Proceedings 83: 1154–1160. 18828976 10.4065/83.10.1154

[20] Gilchrist CA, Houpt E, Trapaidze N, Fei ZJ, Crasta O, et al (2006) Impact of intestinal colonization and invasion on the Entamoeba histolytica transcriptome. Molecular and Biochemical Parasitology 147: 163–176. 16569449 10.1016/j.molbiopara.2006.02.007

[21] Vicente JB, Ehrenkaufer GM, Saraiva LM, Teixeira M, Singh U (2009) Entamoeba histolytica modulates a complex repertoire of novel genes in response to oxidative and nitrosative stresses: implications for amebic pathogenesis. Cell Microbiol 11: 51–69. 18778413 10.1111/j.1462-5822.2008.01236.x PMC3418052

[22] Zhang H, Ehrenkaufer GM, Pompey JM, Hackney JA, Singh U (2008) Small RNAs with 5′-polyphosphate termini associate with a Piwi-related protein and regulate gene expression in the single-celled eukaryote Entamoeba histolytica. PLoS Pathog 4: e1000219. 19043551 10.1371/journal.ppat.1000219 PMC2582682

[23] Zhang H, Pompey JM, Singh U (2011) RNA interference in Entamoeba histolytica: implications for parasite biology and gene silencing. Future Microbiology 6: 103–117. 21162639 10.2217/fmb.10.154 PMC3038252

[24] Ehrenkaufer GM, Haque R, Hackney JA, Eichinger DJ, Singh U (2007) Identification of developmentally regulated genes in Entamoeba histolytica: insights into mechanisms of stage conversion in a protozoan parasite. Cellular Microbiology 9: 1426–1444. 17250591 10.1111/j.1462-5822.2006.00882.x

[25] Mukherjee C, Majumder S, Lohia A, Eichinger D (2009) Inter-Cellular Variation in DNA Content of Entamoeba histolytica Originates from Temporal and Spatial Uncoupling of Cytokinesis from the Nuclear Cycle. PLoS Negl Trop Dis 3: e409. 19352422 10.1371/journal.pntd.0000409 PMC2659751

[26] Singh N, Bhattacharya A, Bhattacharya S (2013) Homologous recombination occurs in Entamoeba and is enhanced during growth stress and stage conversion. PLoS one 8: e74465. 24098652 10.1371/journal.pone.0074465 PMC3787063

[27] Morf L, Singh U (2012) Entamoeba histolytica: a snapshot of current research and methods for genetic analysis. Curr Opin Microbiol 15: 469–475. 22664276 10.1016/j.mib.2012.04.011 PMC3424301

[28] MacFarlane RC, Singh U (2008) Loss of dsRNA-based gene silencing in Entamoeba histolytica: Implications for approaches to genetic analysis. Experimental Parasitology 119: 296–300. 18346737 10.1016/j.exppara.2008.02.001 PMC2426738

[29] Morf L, Pearson RJ, Wang AS, Singh U (2013) Robust gene silencing mediated by antisense small RNAs in the pathogenic protist Entamoeba histolytica. Nucleic Acids Res 41: 9424–9437. 23935116 10.1093/nar/gkt717 PMC3814356

[30] Pearson RJ, Morf L, Singh U (2013) Regulation of H2O2 stress-responsive genes through a novel transcription factor in the protozoan pathogen Entamoeba histolytica. J Biol Chem 288: 4462–4474. 23250742 10.1074/jbc.M112.423467 PMC3567695

[31] Shi H, Tschudi C, Ullu E (2006) An unusual Dicer-like1 protein fuels the RNA interference pathway in Trypanosoma brucei. RNA 12: 2063–2072. 17053086 10.1261/rna.246906 PMC1664728

[32] Bernstein E, Caudy AA, Hammond SM, Hannon GJ (2001) Role for a bidentate ribonuclease in the initiation step of RNA interference. Nature 409: 363–366. 11201747 10.1038/35053110

[33] Grishok A, Pasquinelli AE, Conte D, Li N, Parrish S, et al (2001) Genes and mechanisms related to RNA interference regulate expression of the small temporal RNAs that control C. elegans developmental timing. Cell 106: 23–34. 11461699 10.1016/s0092-8674(01)00431-7

[34] Zhang H, Kolb FA, Jaskiewicz L, Westhof E, Filipowicz W (2004) Single processing center models for human Dicer and bacterial RNase III. Cell 118: 57–68. 15242644 10.1016/j.cell.2004.06.017

[35] Bivalkar-Mehla S, Vakharia J, Mehla R, Abreha M, Kanwar JR, et al (2011) Viral RNA silencing suppressors (RSS): novel strategy of viruses to ablate the host RNA interference (RNAi) defense system. Virus Res 155: 1–9. 20951748 10.1016/j.virusres.2010.10.003 PMC3042272

[36] She X, Xu X, Fedotov A, Kelly WG, Maine EM (2009) Regulation of heterochromatin assembly on unpaired chromosomes during Caenorhabditis elegans meiosis by components of a small RNA-mediated pathway. PLoS Genet 5: e1000624. 19714217 10.1371/journal.pgen.1000624 PMC2726613

[37] Wassenegger M, Heimes S, Riedel L, Sanger HL (1994) RNA-directed de novo methylation of genomic sequences in plants. Cell 76: 567–576. 8313476 10.1016/0092-8674(94)90119-8

[38] Brodersen P, Voinnet O (2006) The diversity of RNA silencing pathways in plants. Trends Genet 22: 268–280. 16567016 10.1016/j.tig.2006.03.003

[39] Vaucheret H (2006) Post-transcriptional small RNA pathways in plants: mechanisms and regulations. Genes Dev 20: 759–771. 16600909 10.1101/gad.1410506

[40] Volpe T, Martienssen RA (2011) RNA interference and heterochromatin assembly. Cold Spring Harb Perspect Biol 3: a003731. 21441597 10.1101/cshperspect.a003731 PMC3181039

[41] Fisher O, Siman-Tov R, Ankri S (2004) Characterization of cytosine methylated regions and 5-cytosine DNA methyltransferase (Ehmeth) in the protozoan parasite Entamoeba histolytica. Nucleic Acids Res 32: 287–297. 14715927 10.1093/nar/gkh161 PMC373271

[42] Ramakrishnan G, Gilchrist CA, Musa H, Torok MS, Grant PA, et al (2004) Histone acetyltransferases and deacetylase in Entamoeba histolytica. Mol Biochem Parasitol 138: 205–216. 15555732 10.1016/j.molbiopara.2004.09.002

[43] Huguenin M, Bracha R, Chookajorn T, Mirelman D (2010) Epigenetic transcriptional gene silencing in Entamoeba histolytica: insight into histone and chromatin modifications. Parasitology 137: 619–627. 19849886 10.1017/S0031182009991363

[44] Diamond LS, Harlow DR, Cunnick CC (1978) A new medium for the axenic cultivation of Entamoeba histolytica and other Entamoeba. Trans R Soc Trop Med Hyg 72: 431–432. 212851 10.1016/0035-9203(78)90144-x

[45] MacFarlane RC, Singh U (2007) Identification of an Entamoeba histolytica serine-, threonine-, and isoleucine-rich protein with roles in adhesion and cytotoxicity. Eukaryotic Cell 6: 2139–2146. 17827347 10.1128/EC.00174-07 PMC2168410

[46] Saito-Nakano Y, Yasuda T, Nakada-Tsukui K, Leippe M, Nozaki T (2004) Rab5-associated Vacuoles play a unique role in phagocytosis of the enteric protozoan parasite Entamoeba histolytica. Journal of Biological Chemistry 279: 49497–49507. 15347665 10.1074/jbc.M403987200

[47] Baxt LA, Rastew E, Bracha R, Mirelman D, Singh U (2010) Downregulation of an Entamoeba histolytica rhomboid protease reveals roles in regulating parasite adhesion and phagocytosis. Eukaryot Cell 9: 1283–1293. 20581296 10.1128/EC.00015-10 PMC2918930

