# Supplementary material for: RNAi Pathway Genes Are Resistant to Small RNA Mediated Gene Silencing in the Protozoan Parasite Entamoeba histolytica
Source: PLoS One. 2014 Sep 8;9(9):e106477. doi: 10.1371/journal.pone.0106477 (PMC4157801; doi:10.1371/journal.pone.0106477)
Supplement: File S1 — Contains the following files: Table S1. Primers used in plasmid construction in this study. A list of all primers used in cloning and PCR probe generation are listed. Table S2. Oligonucleotide probes used in this study. Northern blot oligonucleotide probes are listed. Table S3. RT-PCR primers used in this study. A list of all primers used for RT-PCR. (DOCX) [file pone.0106477.s001.docx]

**Table S1: Primers used in plasmid construction in this study.**

| **Gene Name** | **Gene ID** | **Primer Type** | **Forward Primer Sequence** | **Reverse Primer Sequence** |
| --- | --- | --- | --- | --- |
| EhAgo2-1 | EHI_186850 | Cloning | CCCGGGATGTTAAGTATTTATCC | CTCGAGATAATAAGGATGTGAAC |
| EhAgo2-2 | EHI_125650 | Cloning | CCCGGGCAACATCAATTCACG | CTCGAGTTAGTGATGATGATCT |
| EhAgo2-3 | EHI_177170 | Cloning | CCCGGGATGAAACAAGTTGGAG | CTCGAGATTCATTAGTTCTTC |
| EhRNaseIII | EHI_068740 | Cloning | CCCGGGAGCTCAACTACATTA | CTCGAGTTATTGTGATGGATGAAC |
| EhRdRP1 | EHI_139420 | Cloning | CCCGGGATGGATAAATTTGATTATTGTCAT | CTCGAGTTATTTAATTTGTTTTAGT |
| Ago trigger | EHI_125650 | Cloning/PCR probe | CCTAGGATGCAACCATCAATTCACG | CCTAGGCTTTTTTTGGCAATCTGGTTC |
| Actin | EHI_198930 | PCR probe | CCAGCATCTGAACGTATGGA | CTGGTGCAAGGAATTGTTCA |

**Table S2: Oligonucleotide probes used in this study.**

| **Gene Name** | **Gene ID** | **Probe Sequence** |
| --- | --- | --- |
| EhAgo2-1 | EHI_186850 | ATGTTAAGTATTTATCCAATTAATAATGAT |
| EhAgo2-2 | EHI_125650 | ATGCAACCATCAATTCACGATTACTTCTCA |
| EhAgo2-3 | EHI_177170 | ATGAAACAAGTTGGAGTATTAAGAGACTTC |
| EhRNaseIII | EHI_068740 | ATGAGCTCAACTACATTACACAATGCAATG |
| EhRdRP1 | EHI_139420 | ATGGATAAATTTGATTATTGTCATAAAAAA |
| Trigger | EHI_197520 | ACACTCGAGATGTCTTCAGCTCAACCAACT |
| (Control) | EHI_118130 | GUGGAUAGCCUGGAUAUUGUUGUUGAG |

**Table S3: RT-PCR primers used in this study.**

| **Gene Name** | **Gene ID** | **Forward Primer Sequence** | **Reverse Primer Sequence** |
| --- | --- | --- | --- |
| EhAgo2-1 | EHI_186850 | GGAAGTGGGAATGCAGGATA | AAGAACAGGACGAACAACACC |
| EhAgo2-3 | EHI_177170 | GTTGGGACGATCCTTATCCA | ATATCGGCAGCACGAAAAAC |
| EhRNaseIII | EHI_068740 | TTTGACCTTTTACATTTGGGAGA | GTGGATCAGAACTACTTAATATATA |
| (Control) | EHI_199600 | ACGTCATGCTGAATTTGCTG | CCTTTAAGCCCAGCCTTTCT |
